# T107. WHY VALIDATION MATTERS: A DEMONSTRATION PREDICTING ANTIPSYCHOTIC RESPONSE USING 5 RCTS

**DOI:** 10.1093/schbul/sby016.383

**Published:** 2018-04-01

**Authors:** Adam Chekroud

**Affiliations:** Spring Health

## Abstract

**Background:**

Machine learning methods hold promise for making more effective, personalized treatment decisions to improve outcomes and reduce the cost of care. The use of these techniques remains nascent in psychiatry, and relatively little research has focused on the extent to which models derived in one sample make accurate predictions in unseen samples. Statistical research indicates that model performance in unseen samples is generally lower than performance in the derivation sample.

**Methods:**

We investigate the generalizability of machine learned models using data from five multi-site randomized controlled trials of antipsychotic efficacy (total N = 1511). We include 125 predictor variables collected at baseline in all five trials, including demographics, psychological/behavioral scales (AIMS, BARS, CGI, PANSS, and SARS), vital signs, complete blood count, blood chemistry, and urinalysis. Using elastic net regression, we predicted 4-week treatment outcomes according to a binary cut-point of 25% reduction in PANSS scores. This study compared model performance for a range of internal and external validation methodologies.

**Results:**

First, we trained a separate model on each of the five trials with no internal or external validation and obtained single-trial balanced accuracies from 74.6% to 100%. When each trial was split into a 50% training set and 50% holdout set, the balanced accuracies on the holdout test set were between 48% and 60.6%. When models were trained on each trial using 10-fold cross validation, balanced accuracies ranged from 50% to 73.7%. When each model was trained on a single trial and then sequentially tested on each of the four other trials, the mean balanced accuracy for each trial ranged from 50.5% to 54.2%. Finally, when the model was trained on four trials combined and tested on the one trial left out (leave-one-trial-out validation), balanced accuracies ranged from 48.9% to 58.7%.

**Discussion:**

The performance of models predicting antipsychotic treatment response is highly affected by the validation routine chosen. Performance estimated using one trial – even using internal cross-validation – is drastically higher than the performance obtained when the same model is tested on independent data from other clinical trials with similar protocols. These findings present considerable cause for concern regarding the interpretation of predictive analyses based on a single, multi-site trial.

